# Recent advances in managing lower urinary tract infections

**DOI:** 10.12688/f1000research.16245.1

**Published:** 2018-12-21

**Authors:** Seung-Ju Lee

**Affiliations:** 1 Department of Urology, St. Vincent's Hospital, The Catholic University of Korea, Suwon, South Korea

**Keywords:** Urinary tract infections, antimicrobial resistance, microbiome

## Abstract

Urinary tract infections (UTIs) are among the most common bacterial infections. Traditionally, all symptomatic UTIs are tested and treated. The use of antibiotics has resulted in an antibiotic resistance crisis, and we have limited options for managing UTIs. Currently, we live in the era of antimicrobial resistance and may live in other eras like the era of the microbiome. New insights might provide an opportunity to prevent the overuse and misuse of antibiotics and could enable the development of innovate managing strategies.

## Introduction

The world is changing rapidly; there is hardly anything in life that is not changing. Technological advancements have enriched our lives, but others have created fear and anxiety. The number of antibiotic-resistant pathogens is rapidly increasing, and many pathogens are becoming multidrug resistant (MDR) with the attendant increased risk of failure of standard therapies ^1^. Several decades ago, the first antibiotic saved our lives from bacterial infection, but now bacterial infections have again become a threat. The overuse and misuse of antibiotics, along with the development of few new drugs by the pharmaceutical industry, are the most important causes of the antibiotic resistance crisis ^2^.

Urinary tract infections (UTIs) are among the most common bacterial infections managed by clinicians ^3^. Traditionally, a UTI is defined as microbial infiltration of the normally sterile urinary tract, and most clinicians think all symptomatic UTIs should be treated ^4, 5^. Recently, however, the paradigm for the management of infectious diseases started to shift as the concept of the microbiome has been established ^6^.

## Stop the overuse and misuse of antibiotics

Asymptomatic pyuria and bacteriuria are very common in the elderly, especially in women residing in long-term care facilities or in patients who use a urinary catheter, with a prevalence of 25–50% ^7, 8^. In older residents of long-term care facilities, diagnostic testing and the initiation of antibiotics should be reserved for residents with fever, dysuria, gross hematuria, worsening urinary frequency and incontinence, costovertebral angle tenderness, or suspected bacteremia. Non-specific symptoms and altered mental status are no longer part of the recommended evaluation for a possible UTI ^9, 10^. Asymptomatic bacteriuria also occurs in an estimated 1–5% of healthy pre-menopausal females, increasing to 0.7–27% of patients with diabetes, 2–10% of pregnant women, and 23–89% of patients with spinal cord injuries ^11, 12^. Asymptomatic bacteriuria does not cause systemic disorders, such as renal damage ^13^. Thus, the treatment of asymptomatic bacteriuria is not recommended in patients without risk factors ^11^. Avoiding antibiotic administration in cases where the urine culture does not indicate UTI may be the first step to decreasing antibiotic misuse ^14^.

For patients with non-febrile uncomplicated UTIs, active pain control and minimal use of antibiotics should be prioritized. Uncomplicated cystitis can be a self-limiting disease in some cases. Pain in acute cystitis is a natural consequence of the inflammatory response, and pain-mediated urinary frequency or urgency is the chief complaint of patients. Therefore, for this self-limited disease, painkillers, including NSAIDs, may be a good option for symptomatic care as well as reducing the consumption of antibiotics ^15, 16^. Delaying antibiotic treatment with a back-up prescription to see if symptoms will resolve without antibiotic treatment, or delaying the antibiotic until microbiological results are available, may be an option for antibiotic sparing ^17^.

Recently, multiplex PCR assays for the detection of sexually transmitted infection (STI) agents became commonplace in Eastern Europe, Western Europe, South America, and Asia ^18– 20^. However, the use of unnecessary antibiotics has increased by including strains other than “true” STIs such as *Mycoplasma hominis*, *Ureaplasma urealyticum* (previously *U. urealyticum* biovar 2), and *Ureaplasma parvum* (earlier *U. urealyticum* biovar 1) ^21^. *M. hominis*, *U. urealyticum*, and *U. parvum* are commonly detected in the urogenital tract of both healthy and symptomatic individuals ^22, 23^. Testing for *M. hominis* and *U. parvum* and subsequent antimicrobial treatment of positive men or women are currently not recommended. Instead, “true” STIs and bacterial vaginosis in symptomatic women should be diagnosed and treated ^21^. Bacterial vaginosis, sexually transmitted diseases (STDs), and pelvic inflammatory disease can mimic symptoms of UTIs. In fact, a recent study showed that approximately one-third of STD cases were misdiagnosed as UTIs ^24^.

## The era of antimicrobial resistance

There are limited oral options for the treatment of antibiotic-resistant uropathogens associated with lower UTIs (acute cystitis). Co-trimoxazole was a typical antibiotic used to treat UTIs, but the resistance of *Escherichia coli* to this drug has markedly increased. According to the literature published in the past decade, the resistance rates of *E. coli* to co-trimoxazole varied but were usually over 15–30% ^25– 28^. However, there was an interesting report wherein the authors emphasized the role of co-trimoxazole in empirical antibiotics because of the recent decrease in the resistance rate to co-trimoxazole in several European countries owing to its low prescription rate ^29^. Nevertheless, it may not be possible to reuse the drug worldwide within the next few years, and close observation of surveillance data will be required.

With respect to fluoroquinolones, the increase of resistance in uropathogens has occurred at an alarming rate in relation to increased prescribing practices ^27, 28^. Fluoroquinolones are no longer recommended as first-line therapy for uncomplicated UTIs ^11^. Similar to what was seen with co-trimoxazole, there is evidence that escape from exposure to this antibiotic will increase antimicrobial susceptibility in UTIs. According to Lee *et al*., the susceptibility of Gram-negative bacteria to ciprofloxacin was much higher in patients younger than 20 years old than in patients older than 20 years old. The reason for this observation may be the lower exposure to fluoroquinolones in young individuals because these drugs are not recommended for use in those under 20 years old ^30^.

One recent issue of importance is the increasing prevalence of extended spectrum beta lactamase (ESBL)-producing uropathogens. Before 2010, the vast majority of countries showed less than a 5–10% prevalence of ESBL-producing *E. coli*, whereas the prevalence exceeded 10% in the local communities of many countries after 2010 ^31– 35^. Therefore, the increase in ESBL-producing *E. coli* is no different to that of co-trimoxazole-resistant *E. coli* or fluoroquinolone-resistant *E. coli*, and the prevalence of ESBL-producing *E. coli* is likely to increase soon.

## Use of re-emerging older antibiotics

Fosfomycin is an oral antibiotic agent that has broad activity against MDR uropathogens including ESBL-producing *E. coli*. Fosfomycin prevents peptidoglycan synthesis earlier than do beta-lactam or glycopeptide antibiotics and is broadly active against several Gram-positive and Gram-negative organisms, including methicillin-resistant *Staphylococcus aureus* and vancomycin-resistant *Enterococcus* spp.. Fosfomycin has been shown to have advantages in the treatment of UTIs owing to its high concentration in the urinary tract, which exceeds 2,000 mg/L after the initial administration and remains at high levels for a prolonged period, over 24 hours. However, oral fosfomycin should not be used for pyelonephritis or in patients with bacteremia because of inadequate concentrations within the bloodstream ^36, 37^. Fosfomycin susceptibility in uropathogens, including *E. coli*, is currently greater than 90%, even in ESBL-producing *E. coli* ^38– 40^. It is safe to use in pregnancy ^41^. The drug is likely to be excreted in low levels in breast milk, but would not be expected to cause any adverse effects in breastfed infants, especially if the infant is older than two months. ^42^.

Another oral antimicrobial agent that can be considered for the treatment of ESBL-producing *E. coli* cystitis is nitrofurantoin. Nitrofurantoin is a drug that has been used since the 1950s to treat uncomplicated UTIs and works by damaging bacterial DNA in its highly active reduced form. Now, and even in earlier eras of widespread use, the baseline resistance to nitrofurantoin is low (0–10%) ^43, 44^. Nitrofurantoin should be used only for lower UTIs, and its use should be avoided in patients with a creatinine clearance of less than 60 mL/minute, as reduced renal function results in decreased active drug within the urine ^45^. Nitrofurantoin is one of the few drugs that can be used during pregnancy ^46^. A recent retrospective, matched-cohort study in older adults concluded that long-term use of nitrofurantoin is associated with greater risk of lung injury than acute exposure ^47^.

## Role for non-antimicrobial prophylaxis

The active use of non-antimicrobial prophylaxis is often indicated and does not result in an increase in antimicrobial resistance of the commensal flora. Immunoactive agents, probiotics ( *Lactobacillus* spp.), cranberry-based products, D-mannose, methenamine hippurate, hormonal replacement (in post-menopausal women), and other options have been studied as non-antimicrobial prophylaxis ^48– 53^. Evidence for the use of non-antimicrobial prophylaxis is hampered by considerable heterogeneity, and further placebo-controlled randomized trials of these agents are needed. However, trials investigating these options have produced promising results and combining these agents may offer the best route to lowering the rate of recurrent UTIs without needing to use antimicrobials ^54^.

Among these modalities, the urinary immunopotentiator is now well documented and strongly recommended in the guidelines ^11^. The oral immunostimulant OM-89 (Uro-Vaxom ^®^), an extract of 18 different serotypes of heat-killed uropathogenic *E. coli*, stimulates innate immunity by increasing non-specific and specific humoral and cellular immune responses via the induction of interferon-γ and tumor necrosis factor-γ production as well as the activities of lymphocytes and macrophages ^55– 57^. Uro-Vaxom ^®^ is a safe and effective medicine that can reduce recurrent UTI episodes ^48, 58– 60^ and can effectively reduce the repeated use of antibiotics ^61^. Uromune ^®^ is a sublingual spray consisting of equal amounts of four common UTI-causing bacteria in a suspension of 10 ^9^ inactivated whole bacteria/mL: *E. coli*; *Klebsiella pneumoniae*, *Proteus vulgaris*, and *Enterococcus faecalis* ^54^. The data from European prospective and retrospective studies suggest that Uromune ^®^ is a viable alternative therapy for treating recurrent UTIs in women ^62, 63^.

The lower estrogen state found in postmenopausal women is linked with decreased innate immunity via the loss of the commensal bacteria *Lactobacillus* and the loss of the acidic pH microenvironment within the vagina. Although specific mechanisms are still poorly understood, estrogen plays a key role in modulating the natural defense of the lower urinary tract against UTIs ^64^. The role for topical (intravaginal cream, vaginal rings, impregnated pessary rings, vaginal pessaries, and vaginal tablets) estrogen in averting recurrent UTIs in postmenopausal women compared with placebo is clear, and guidelines for this population recommend their use ^11, 65, 66^. Currently, there is evidence that CO _2_ ablative vaginal lasers may help rejuvenate this microenvironment, much like topical estrogen therapy, restoring the lactic acid synthesis of commensal bacteria and the innate vaginal defense against UTIs ^67– 69^. For postmenopausal women, vaginal estrogen therapy has been considered often as an adjunct to antimicrobial-based prophylaxis ^70^. However, combination therapy with both immunostimulants and vaginal therapy (laser rejuvenation or estrogen) may provide better effectiveness at preventing UTI recurrence in postmenopausal women and is a potentially novel avenue for further research.

## Paradigm shifting in the era of the microbiome

The microbiota is defined as the microorganisms in a particular environment. The microbiome refers to their genomes that are revealed using molecular techniques such as 16S ribosomal RNA (rRNA) sequencing ^71^. Recently, more sensitive diagnostic tests demonstrated that urine is not sterile ^72^. The urinary tract is inhabited by a unique urinary microbiota, and standard bacteriuria represents a fraction of the diverse microbiota hosted by the urinary tract ^73^. In the past, the notion that UTI was the detection of organisms as a standard culture in sterile urine has changed in the era of the microbiome. The fact that we diagnosed UTIs through standard culture and antibiotic susceptibility ignored the dozens of bacterial species and intracellular bacterial colonies known to reside in the urinary tract ^72^. As expected, in the era of the microbiome, stable bacterial communities are generally beneficial and this condition is referred to as symbiosis. In this sense, patients with urinary tract symptoms would be more likely to be classified as having urinary tract dysbiosis rather than a UTI ^73^. If the full array of microbes resident in the human urinary tract is identified in the near future, some treatment of UTIs by antibiotics may turn into a correction of dysbiosis. In the gut microbiome, treatments of *Clostridium difficile* infections through fecal transplants have been investigated ^74, 75^. In the urinary tract, the instillation of non-pathogenic *E. coli* safely reduced the risk of symptomatic UTI in patients with spinal cord injury. All of these efforts are ultimately related to preventing the overuse and misuse of antibiotics. The paradigm of managing UTIs has shifted from diagnosing UTIs and antibiotic treatment to screening for patients who really need antibiotics.

## Conclusions

A clear achievement in recent oncology is the emergence of immunotherapy, a type of treatment that helps our immune system fight cancer and that marks an entirely different way of treating cancer by targeting the immune system, not the tumor itself. If the immune system also helps our body fight infections, why not use our body’s immune system instead of antibiotics to treat UTIs? This paradigm shift seems to be beginning in the management of UTIs. Further understanding of the microbiome in the urogenital system is expected to stimulate this shift. Eventually, the spread of antibiotic resistance can be reduced if antibiotics are used only when they are needed.

## References

[1] Tillotson GS Zinner SH: Burden of antimicrobial resistance in an era of decreasing susceptibility. *Expert Rev Anti Infect Ther.* 2017;15(7):663–76. 10.1080/14787210.2017.1337508 28580804

[2] Morehead MS Scarbrough C: Emergence of Global Antibiotic Resistance. *Prim Care.* 2018;45(3):467–84. 10.1016/j.pop.2018.05.006 30115335

[3] Tandogdu Z Wagenlehner FM: Global epidemiology of urinary tract infections. *Curr Opin Infect Dis.* 2016;29(1):73–9. 10.1097/QCO.0000000000000228 26694621

[4] Barber AE Norton JP Spivak AM: Urinary tract infections: current and emerging management strategies. *Clin Infect Dis.* 2013;57(5):719–24. 10.1093/cid/cit284 23645845 PMC3739462

[5] Sobel JD: Urinary tract infections.In: Mandell GL, Douglas RG Jr, Bennett JE, Dolin R, eds. *Mandell, Douglas, and Bennett’s Principles and Practice of Infectious Diseases* 7th ed. Philadelphia, PA: Elsevier Churchill Livingstone;2009:957–985.

[6] Whiteside SA Razvi H Dave S: The microbiome of the urinary tract--a role beyond infection. *Nat Rev Urol.* 2015;12(2):81–90. 10.1038/nrurol.2014.361 25600098

[7] Hooton TM Bradley SF Cardenas DD: Diagnosis, prevention, and treatment of catheter-associated urinary tract infection in adults: 2009 International Clinical Practice Guidelines from the Infectious Diseases Society of America. *Clin Infect Dis.* 2010;50(5):625–63. 10.1086/650482 20175247

[8] Ducharme J Neilson S Ginn JL: Can urine cultures and reagent test strips be used to diagnose urinary tract infection in elderly emergency department patients without focal urinary symptoms? *CJEM.* 2007;9(2):87–92. 10.1017/S1481803500014846 17391578

[9] Yin P Kiss A Leis JA: Urinalysis Orders Among Patients Admitted to the General Medicine Service. *JAMA Intern Med.* 2015;175(10):1711–3. 10.1001/jamainternmed.2015.4036 26280990

[10] Daley P Penney C Wakeham S: Urinary tract infection diagnosis and response to therapy in long-term care: A prospective observational study. *Can J Infect Dis Med Microbiol.* 2015;26(3):133–6. 10.1155/2015/830415 26236354 PMC4507838

[11] Bonkat G Pickard R Bartoletti R: European Association of Urology (EAU) Guidelines Office.Guidelines on urological infections.2017;8–9.

[12] Nicolle LE Bradley S Colgan R: Infectious Diseases Society of America guidelines for the diagnosis and treatment of asymptomatic bacteriuria in adults. *Clin Infect Dis.* 2005;40(5):643–54. 10.1086/427507 15714408

[13] Tencer J: Asymptomatic bacteriuria--a long-term study. *Scand J Urol Nephrol.* 1988;22(1):31–4. 10.1080/00365599.1988.11690380 3387908

[14] Lee MJ Kim M Kim NH: Why is asymptomatic bacteriuria overtreated?: A tertiary care institutional survey of resident physicians. *BMC Infect Dis.* 2015;15:289. 10.1186/s12879-015-1044-3 26209977 PMC4514993

[15] Vik I Bollestad M Grude N: Ibuprofen versus mecillinam for uncomplicated cystitis--a randomized controlled trial study protocol. *BMC Infect Dis.* 2014;14:693. 10.1186/s12879-014-0693-y 25516016 PMC4273435

[16] Gágyor I Bleidorn J Kochen MM: Ibuprofen versus fosfomycin for uncomplicated urinary tract infection in women: randomised controlled trial. *BMJ.* 2015;351:h6544. 10.1136/bmj.h6544 26698878 PMC4688879

[17] Little P Moore MV Turner S: Effectiveness of five different approaches in management of urinary tract infection: randomised controlled trial. *BMJ.* 2010;340:c199. 10.1136/bmj.c199 20139214 PMC2817051

[18] Kim Y Kim J Lee KA: Prevalence of sexually transmitted infections among healthy Korean women: implications of multiplex PCR pathogen detection on antibiotic therapy. *J Infect Chemother.* 2014;20(1):74–6. 10.1016/j.jiac.2013.08.005 24462432

[19] Fernández G Martró E González V: Usefulness of a novel multiplex real-time PCR assay for the diagnosis of sexually-transmitted infections. *Enferm Infecc Microbiol Clin.* 2016;34(8):471–6. 10.1016/j.eimc.2015.10.014 26706392

[20] Del Prete R Ronga L Lestingi M: Simultaneous detection and identification of STI pathogens by multiplex Real-Time PCR in genital tract specimens in a selected area of Apulia, a region of Southern Italy. *Infection.* 2017;45(4):469–77. 10.1007/s15010-017-1002-7 28260146

[21] Horner P Donders G Cusini M: Should we be testing for urogenital *Mycoplasma hominis*, *Ureaplasma parvum* and *Ureaplasma urealyticum* in men and women? - a position statement from the European STI Guidelines Editorial Board. *J Eur Acad Dermatol Venereol.* 2018;32(11):1845–51. 10.1111/jdv.15146 29924422

[22] Robertson JA Stemke GW Davis JW Jr: Proposal of Ureaplasma parvum sp. nov. and emended description of Ureaplasma urealyticum (Shepard *et al.* 1974) Robertson *et al.* 2001. *Int J Syst Evol Microbiol.* 2002;52(Pt 2):587–97. 10.1099/00207713-52-2-587 11931172

[23] Taylor-Robinson D: Mollicutes in vaginal microbiology: *Mycoplasma hominis*, *Ureaplasma urealyticum*, *Ureaplasma parvum* and *Mycoplasma genitalium*. *Res Microbiol.* 2017;168(9–10):875–81. 10.1016/j.resmic.2017.02.009 28263902

[24] Tomas ME Getman D Donskey CJ: Overdiagnosis of Urinary Tract Infection and Underdiagnosis of Sexually Transmitted Infection in Adult Women Presenting to an Emergency Department. *J Clin Microbiol.* 2015;53(8):2686–92. 10.1128/JCM.00670-15 26063863 PMC4508438

[25] Yang B Yang F Wang S: Analysis of the spectrum and antibiotic resistance of uropathogens in outpatients at a tertiary hospital. *J Chemother.* 2018;30(3):145–9. 10.1080/1120009X.2017.1418646 29304717

[26] Seo MR Kim SJ Kim Y: Susceptibility of *Escherichia coli* from community-acquired urinary tract infection to fosfomycin, nitrofurantoin, and temocillin in Korea. *J Korean Med Sci.* 2014;29(8):1178–81. 10.3346/jkms.2014.29.8.1178 25120333 PMC4129215

[27] Chervet D Lortholary O Zahar JR: Antimicrobial resistance in community-acquired urinary tract infections in Paris in 2015. *Med Mal Infect.* 2018;48(3):188–92. 10.1016/j.medmal.2017.09.013 29054298

[28] Seitz M Stief C Waidelich R: Local epidemiology and resistance profiles in acute uncomplicated cystitis (AUC) in women: a prospective cohort study in an urban urological ambulatory setting. *BMC Infect Dis.* 2017;17(1):685. 10.1186/s12879-017-2789-7 29037164 PMC5644167

[29] Caron F Wehrle V Etienne M: The comeback of trimethoprim in France. *Med Mal Infect.* 2017;47(4):253–60. 10.1016/j.medmal.2016.12.001 28043762

[30] Lee DS Choe HS Kim HY: Role of age and sex in determining antibiotic resistance in febrile urinary tract infections. *Int J Infect Dis.* 2016;51:89–96. 10.1016/j.ijid.2016.08.015 27575938

[31] Toner L Papa N Aliyu SH: Extended-spectrum beta-lactamase-producing Enterobacteriaceae in hospital urinary tract infections: incidence and antibiotic susceptibility profile over 9 years. *World J Urol.* 2016;34(7):1031–7. 10.1007/s00345-015-1718-x 26511749

[32] Arpin C Quentin C Grobost F: Nationwide survey of extended-spectrum {beta}-lactamase-producing Enterobacteriaceae in the French community setting. *J Antimicrob Chemother.* 2009;63(6):1205–14. 10.1093/jac/dkp108 19329798

[33] Martin D Fougnot S Grobost F: Prevalence of extended-spectrum beta-lactamase producing Escherichia coli in community-onset urinary tract infections in France in 2013. *J Infect.* 2016;72(2):201–6. 10.1016/j.jinf.2015.11.009 26702736

[34] Hoban DJ Nicolle LE Hawser S: Antimicrobial susceptibility of global inpatient urinary tract isolates of *Escherichia coli*: results from the Study for Monitoring Antimicrobial Resistance Trends (SMART) program: 2009-2010. *Diagn Microbiol Infect Dis.* 2011;70(4):507–11. 10.1016/j.diagmicrobio.2011.03.021 21767706

[35] Doi Y Park YS Rivera JI: Community-associated extended-spectrum β-lactamase-producing *Escherichia coli* infection in the United States. *Clin Infect Dis.* 2013;56(5):641–8. 10.1093/cid/cis942 23150211 PMC3563390

[36] Raz R: Fosfomycin: an old--new antibiotic. *Clin Microbiol Infect.* 2012;18(1):4–7. 10.1111/j.1469-0691.2011.03636.x 21914036

[37] Naber KG Thyroff-Friesinger U: Fosfomycin trometamol versus ofloxacin/co-trimoxazole as single dose therapy of acute uncomplicated urinary tract infection in females: a multicentre study. *Infection.* 1990;18 Suppl 2:S70–6. 10.1007/BF01643431 2286465

[38] Oteo J Orden B Bautista V: CTX-M-15-producing urinary *Escherichia coli* O25b-ST131-phylogroup B2 has acquired resistance to fosfomycin. *J Antimicrob Chemother.* 2009;64(4):712–7. 10.1093/jac/dkp288 19671590

[39] Honderlick P Cahen P Gravisse J: [Uncomplicated urinary tract infections, what about fosfomycin and nitrofurantoin in 2006?]. *Pathol Biol (Paris).* 2006;54(8–9):462–6. 10.1016/j.patbio.2006.07.016 17027182

[40] Cho YH Jung SI Chung HS: Antimicrobial susceptibilities of extended-spectrum beta-lactamase-producing *Escherichia coli* and *Klebsiella pneumoniae* in health care-associated urinary tract infection: focus on susceptibility to fosfomycin. *Int Urol Nephrol.* 2015;47(7):1059–66. 10.1007/s11255-015-1018-9 26026972

[41] Deck D Winston L: Beta-Lactam and other cell wall- and membrane-active antibiotics.In: *Basic & clinical pharmacology* 12th edition. New York: McGraw-Hill;2012.

[42] Deck D Winston L: Sulfonamides, trimethoprim, quinolones.In: *Basic and clinical pharmacology* 12th edition. New York: McGraw-Hill;2012.

[43] Melekos MD Naber KG: Complicated urinary tract infections. *Int J Antimicrob Agents.* 2000;15(4):247–56. 10.1016/S0924-8579(00)00168-0 10929873

[44] Huttner A Verhaegh EM Harbarth S: Nitrofurantoin revisited: a systematic review and meta-analysis of controlled trials. *J Antimicrob Chemother.* 2015;70(9):2456–64. 10.1093/jac/dkv147 26066581

[45] Oplinger M Andrews CO: Nitrofurantoin contraindication in patients with a creatinine clearance below 60 mL/min: Looking for the evidence. *Ann Pharmacother.* 2013;47(1):106–11. 10.1345/aph.1R352 23341159

[46] Lee M Bozzo P Einarson A: Urinary tract infections in pregnancy. *Can Fam Physician.* 2008;54(6):853–4. 18556490 PMC2426978

[47] Santos JM Batech M Pelter MA: Evaluation of the Risk of Nitrofurantoin Lung Injury and Its Efficacy in Diminished Kidney Function in Older Adults in a Large Integrated Healthcare System: A Matched Cohort Study. *J Am Geriatr Soc.* 2016;64(4):798–805. 10.1111/jgs.14072 27100576

[48] Beerepoot MAJ Geerlings SE van Haarst EP: Nonantibiotic prophylaxis for recurrent urinary tract infections: a systematic review and meta-analysis of randomized controlled trials. *J Urol.* 2013;190(6):1981–9. 10.1016/j.juro.2013.04.142 23867306

[49] Schwenger EM Tejani AM Loewen PS: Probiotics for preventing urinary tract infections in adults and children. *Cochrane Database Syst Rev.* 2015; (12):CD008772. 10.1002/14651858.CD008772.pub2 26695595 PMC8720415

[50] Kontiokari T Sundqvist K Nuutinen M: Randomised trial of cranberry-lingonberry juice and *Lactobacillus* GG drink for the prevention of urinary tract infections in women. *BMJ.* 2001;322(7302):1571. 10.1136/bmj.322.7302.1571 11431298 PMC33514

[51] Kranjčec B Papeš D Altarac S: D-mannose powder for prophylaxis of recurrent urinary tract infections in women: a randomized clinical trial. *World J Urol.* 2014;32(1):79–84. 10.1007/s00345-013-1091-6 23633128

[52] Lo TS Hammer KD Zegarra M: Methenamine: a forgotten drug for preventing recurrent urinary tract infection in a multidrug resistance era. *Expert Rev Anti Infect Ther.* 2014;12(5):549–54. 10.1586/14787210.2014.904202 24689705

[53] Raz R Stamm WE: A controlled trial of intravaginal estriol in postmenopausal women with recurrent urinary tract infections. *N Engl J Med.* 1993;329(11):753–6. 10.1056/NEJM199309093291102 8350884

[54] Sihra N Goodman A Zakri R: Nonantibiotic prevention and management of recurrent urinary tract infection. *Nat Rev Urol.* 2018;15(12):750–776. 10.1038/s41585-018-0106-x 30361493

[55] Wybran J Libin M Schandene L: Activation of natural killer cells and cytokine production in man by bacterial extracts. *Immunopharmacol Immunotoxicol.* 1989;11(1):17–32. 10.3109/08923978909082140 2503554

[56] van Pham T Kreis B Corradin-Betz S: Metabolic and functional stimulation of lymphocytes and macrophages by an *Escherichia coli* extract (OM-89): *in vitro* studies. *J Biol Response Mod.* 1990;9(2):231–40. 2160522

[57] Bosch A Benedi VJ Pares R: Enhancement of the humoral immune response and resistance to bacterial infection in mice by the oral administration of a bacterial immunomodulator (OM-89). *Immunopharmacol Immunotoxicol.* 1988;10(3):333–43. 10.3109/08923978809041425 3143754

[58] Bauer HW Rahlfs VW Lauener PA: Prevention of recurrent urinary tract infections with immuno-active *E. coli* fractions: a meta-analysis of five placebo-controlled double-blind studies. *Int J Antimicrob Agents.* 2002;19(6):451–6. 10.1016/S0924-8579(02)00106-1 12135831

[59] Naber KG Cho YH Matsumoto T: Immunoactive prophylaxis of recurrent urinary tract infections: a meta-analysis. *Int J Antimicrob Agents.* 2009;33(2):111–9. 10.1016/j.ijantimicag.2008.08.011 18963856

[60] Bauer HW Alloussi S Egger G: A long-term, multicenter, double-blind study of an *Escherichia coli* extract (OM-89) in female patients with recurrent urinary tract infections. *Eur Urol.* 2005;47(4):542–8; discussion 548. 10.1016/j.eururo.2004.12.009 15774256

[61] Muzzi-Bjornson L Macera L: Preventing infection in elders with long-term indwelling urinary catheters. *J Am Acad Nurse Pract.* 2011;23(3):127–34. 10.1111/j.1745-7599.2010.00588.x 21355945

[62] Lorenzo-Gómez MF Padilla-Fernández B García-Cenador MB: Comparison of sublingual therapeutic vaccine with antibiotics for the prophylaxis of recurrent urinary tract infections. *Front Cell Infect Microbiol.* 2015;5:50. 10.3389/fcimb.2015.00050 26090341 PMC4452880

[63] Yang B Foley S: First experience in the UK of treating women with recurrent urinary tract infections with the bacterial vaccine Uromune ^®^. *BJU Int.* 2018;121(12):289–92. 10.1111/bju.14067 29171130

[64] Hannan TJ Hooton TM Hultgren SJ: Estrogen and recurrent UTI: what are the facts? *Sci Transl Med.* 2013;5(190):190fs23. 10.1126/scitranslmed.3006423 23785033

[65] Perrotta C Aznar M Mejia R: Oestrogens for preventing recurrent urinary tract infection in postmenopausal women. *Cochrane Database Syst Rev.* 2008; (2):CD005131. 10.1002/14651858.CD005131.pub2 18425910 PMC13598824

[66] Suckling J Lethaby A Kennedy R: Local oestrogen for vaginal atrophy in postmenopausal women. *Cochrane Database Syst Rev.* 2006; (4):CD001500. 10.1002/14651858.CD001500.pub2 17054136

[67] Salvatore S Leone Roberti Maggiore U Athanasiou S: Histological study on the effects of microablative fractional CO2 laser on atrophic vaginal tissue: an *ex vivo* study. *Menopause.* 2015;22(8):845–9. 10.1097/GME.0000000000000401 25608269

[68] Zerbinati N Serati M Origoni M: Microscopic and ultrastructural modifications of postmenopausal atrophic vaginal mucosa after fractional carbon dioxide laser treatment. *Lasers Med Sci.* 2015;30(1):429–36. 10.1007/s10103-014-1677-2 25410301

[69] YANG BOB Foley S: MP23-05 USING FEMTOUCH™ IN THE TREATMENT OF WOMEN WITH RECURRENT URINARY TRACT INFECTIONS: FIRST EXPERIENCE IN THE UNITED KINGDOM. *J Urol.* 2017;197(4):e294–e295. 10.1016/j.juro.2017.02.733

[70] Hooton TM Gupta K: UpToDate.Waltham, MA: [accessed 5 June 2016]. Recurrent urinary tract infection in women.2016.

[71] Ursell LK Metcalf JL Parfrey LW: Defining the human microbiome. *Nutr Rev.* 2012;70 Suppl 1:S38–44. 10.1111/j.1753-4887.2012.00493.x 22861806 PMC3426293

[72] Hilt EE McKinley K Pearce MM: Urine is not sterile: use of enhanced urine culture techniques to detect resident bacterial flora in the adult female bladder. *J Clin Microbiol.* 2014;52(3):871–6. 10.1128/JCM.02876-13 24371246 PMC3957746

[73] Finucane TE: 'Urinary Tract Infection' and the Microbiome. *Am J Med.* 2017;130(3):e97–e98. 10.1016/j.amjmed.2016.08.018 27593612

[74] Vrieze A Van Nood E Holleman F: Transfer of intestinal microbiota from lean donors increases insulin sensitivity in individuals with metabolic syndrome. *Gastroenterology.* 2012;143(4):913–6.e7. 10.1053/j.gastro.2012.06.031 22728514

[75] Youngster I Sauk J Pindar C: Fecal microbiota transplant for relapsing *Clostridium difficile* infection using a frozen inoculum from unrelated donors: a randomized, open-label, controlled pilot study. *Clin Infect Dis.* 2014;58(11):1515–22. 10.1093/cid/ciu135 24762631 PMC4017893

